# Midwives’ knowledge and practice of Respectful Maternity Care: a survey from Iran

**DOI:** 10.1186/s12884-022-05065-4

**Published:** 2022-10-05

**Authors:** Maryam Moridi, Farzaneh Pazandeh, Barbara Potrata

**Affiliations:** 1 Department of Midwifery and Reproductive Health, School of Nursing and Midwifery, Tehran University of Medical Sciences, Tehran, Iran; 2 School of Health Sciences, University of Nottingham, Nottingham, UK; 3 Independent Scholar, Rotterdam, The Netherlands

**Keywords:** Respect, Maternity care, Knowledge, Practice, Midwifery

## Abstract

**Background:**

In the past decade, countries worldwide aimed to evaluate the quality of childbirth care and reduce the high rates of disrespect and abuse during childbirth. Few studies have attempted to identify providers’ characteristics associated with respectful maternity care quantitatively. This study aims to evaluate midwives’ knowledge and practice of respectful maternity care (RMC).

**Methods:**

A cross-sectional study was carried out in 15 teaching and non-teaching hospitals in Tehran, Iran. The hospitals were selected by using a cluster sampling design. Midwives’ Knowledge and Practice of Respectful Maternity Care scale (MKP-RMC) was administered to 250 midwives working in maternity units at study hospitals. The data were analysed by statistics package for social science (SPSS, version 21.0, Chicago, IL).

**Results:**

Findings demonstrated that the mean score for knowledge and practice of midwives were 20.96 ± 3.54 and 101.64 ± 11.49, respectively. Also, in both knowledge and practice scales, midwives had the highest score in “providing safe care’ domain and the lowest score in “preventing mistreatment” domain.

**Conclusion:**

Our findings showed that for Iranian midwives, providing care to preserve mothers’ and their babies’ wellbeing is more critical than preventing maternal mistreatment, resulting from the importance of the care provision in the Iranian healthcare system. Promoting midwives’ knowledge and practice through developing a tailored educational program to prevent mistreatment and providing emotional support alongside physical care is recommended.

## Introduction

There is a consensus that disrespectful care during childbirth decreases women’s satisfaction with maternity services and is a key barrier to seeking facility-based maternity care for future births. [1–3] The United Nation adopted seventeen sustainable development goals in 2015 and the third goal is “to ensure healthy lives and promote wellbeing for all at all ages”. [4] Therefore, ensuring facility-based RMC is essential for improving maternal and neonatal health, especially in low- and middle- income countries where maternal mortality and non-skilled delivery care remain high. The White Ribbon Alliance (WRA) developed seven domains of the Universal Rights of Childbearing Women Charter. [5] The World Health Organization (WHO) also advocates implementing evidence-based and Respectful Maternity Care (RMC) that values women’s individual, cultural, personal, and medical needs as an essential strategy for universal access to high-quality care. [6].

In the past decade, efforts have been made to document different aspects and extent of disrespect and abuse during childbirth. [3, 7] Several qualitative studies have identified potential factors associated with a higher risk of disrespect and abuse from laboring women’s point of view, including race/ethnicity and religion; [8, 9] being young, unmarried woman, [10] high parity [11] and having at-risk pregnancy. [12, 13] Although the evaluation of disrespect and abuse is essential, it is necessary to improve the quality of maternity care to achieve women-centred and respectful care. Understanding the facilitators of and barriers to implementing RMC is critical to promoting RMC in these contexts. [14] The health care providers’ characteristics and perspectives of respectful care during childbirth are an essential component for improving the quality of care. [15] Midwives are skilled, knowledgeable, and compassionate caregivers for childbearing women, newborns and families throughout pre-pregnancy, pregnancy, birth, postpartum and the early weeks of life. [16] Midwives are the principal caregivers and the backbone of maternity services. [17] Globally, midwives and the childbirth care that they provide have an important role in different societies, and they are the key actors in change and promoting RMC. [18].

In Iran, the majority of births take place in maternity services, where the medical model of care is dominant and midwives work under the supervision of obstetricians. The mothers’ Bill of Rights was developed in 2003 to support the laboring women’s rights [19] and respecting laboring women has been included in Iran’s National Guidelines for Normal Childbirth [20]. However, previous studies have demonstrated that the principles of RMC are frequently not implemented appropriately in practice. Studies exploring Iranian women’s experiences reported that the quality of childbirth care is not optimal. For example, some women did not have access to basic birth facilities and did not receive timely prevention and detection of complications during birth. Some women did not receive enough support, continuity of care, respect, and safety. [9, 21, 22]. In addition, in a qualitative study, midwives defined RMC as showing empathy, providing women-centred care and protecting rights; it could be concluded that Iranian midwives know the RMC’s components sufficiently. [23] This study aimed to evaluate midwives’ knowledge and practice of respectful maternity care (RMC).

## Methods and materials

### Study design and participants

This was a cross-sectional survey that was carried out in fifteen hospitals in the four regions of Tehran, Iran, from January to July 2019. These hospitals provide childbirth care for women from low-middle income families and are supervised by three medical universities in Tehran and the Iranian Social Security Organization. The participants were 250 midwives with an academic degree and had been working in the labor and birth units for more than a year.

### Sample size

The sample size was determined based on the Cochrane formula and using a previous study’s findings about midwives’ knowledge (18.8%).^16^ The sample size was determined to be 250 to detect a medium-sized effect, with 95% power at a significant level of 5%, assuming a precision of 0.05.

### Study questionnaires

This study employed two questionnaires: a questionnaire for recording demographic and obstetric characteristics and the Midwives’ Knowledge and Practice of Respectful Maternity Care scale (MKP-RMC). The MKP-RMC was designed and assessed for validity and reliability in Tehran, Iran and showed appropriate validity and reliability. [24] The Content Validity Index (CVI) and Content Validity Ratio (CVR) of the MKP-RMC was above 0.9. The knowledge and practice sections of MKP-RMC scale had good internal consistency (0.72 and 0.95, respectively). The intra-class correlation coefficients in knowledge and practice sections were 0.92 and 0.79, respectively indicating an appropriate stability of the scale. This scale has two sections with 23 items in each for evaluating knowledge and practice of midwives. There are three domains in both knowledge and practice sections including: “giving emotional support” (12 and 11 items, respectively), “providing safe care” (8 and 9 items, respectively) and “preventing mistreatment” (3 and 3 items, respectively). Moridi et al., (2020) described the items and method of scoring of MKP-RMC.

To assess the midwives’ knowledge, each item was dichotomized, with 1 representing agreement and 0 representing disagreement with the item. The practice scale consisted of items originally assessed on a five-point Likert scale (always, often, sometimes, rarely, and never). A composite score was then created by summing all the individual items within each scale. As the subscales had different ranges, they were transformed to standard range of 0 to 100 calculating [the sum of obtained score (x) – min (x) / max (x) – min (x)] × 100. The score of 0 being the lowest, and 100 being the highest score possible. For performing the logistic regression, the total scores of knowledge and practice (as dependent variables) were dichotomized to satisfactory and unsatisfactory. The scores below percentile 50 were defined as unsatisfactory and those above it as satisfactory. The median of the knowledge and practice scales was 22 and 104 respectively. The significant level was set as *p* < 0.05.

### Procedure

First, the study protocol was approved by research committee of Shahid Beheshti University of Medical Sciences. Permission for the study was then obtained from the study hospitals’ officials and ethic committees. Next, the informed consent was signed by participating midwives. The fifteen hospitals which participated in this study were selected randomly from the teaching, non-teaching and semi-private hospitals in Tehran. Cluster sampling was used for selection of these hospitals. First, fifteen districts were selected from all 22 districts of Tehran, randomly. Then, one hospital in each district was selected randomly and all eligible midwives in these hospitals were invited to participate. Participant midwives completed the self-administered questionnaire in a quiet room in a birthing unit at the time convenient to them.

### Statistical analysis

The data were entered into statistics package for social science (SPSS, version 21.0, Chicago, IL) and tested for normality. Descriptive statistics including frequency, percentage, mean, and standard deviations were used. The forward Logistic Regression Model was developed to identify the association between knowledge and practice total scores (as dependent variable) and demographic and obstetric characteristics (as independent variable).

## Results

### Socio-demographic characteristics

This study recruited 250 eligible midwives. Most midwives were between 26 and 35 years old (44%) and the mean (SD) age was 33.30 (8.75) years. Most midwives had a bachelor’s degree (80.40%), work experience of less than 10 years (70%) and were employed on a temporary contract (61.60%). The majority of participating midwives were married (60%) and had no children (62%) (Table1).

**Table 1:** The characteristics of the participant midwives

Variables	n = 250
Age (years) ≤ 25 26–35 36–45 > 46 Mean(SD)	49 (19.6) 110 (44) 61 (24.40) 30 (12) 33.30 (8.75)
Marital status Single Married	100 (40) 150 (60)
Education [n (%)] Associate Degree Bachelor MSc	8 (3.20) 201 (80.40) 41 (16.40)
Work experience (year) < 10 10–20 > 20 Mean (SD)	175 (70) 48 (19.20) 27 (10.80) 8.46 (7.68)
Having children Yes No	95 (38) 155 (62)
Employment Permanent Temporary	96 (38.40) 154 (61.60)

### Midwives’ knowledge and practice of RMC score

The overall mean (SD) scores of midwives’ knowledge and practice were 89.90 (± 3.54) and 85.47 (± 11.49) respectively which indicates that the midwives had a relatively good knowledge and practice on RMC.

The highest mean scores in the knowledge (93.8) and practice (90.4) were in the ‘providing safe care’ domain, respectively. The lowest mean score of midwives’ knowledge (78) and practice (68.3) were in the ‘preventing maltreatment’ domain, respectively (Table2).

**Table 2:** Midwives knowledge and practice of RMC in related domains

Domains	Knowledge	Practice
	Mean ± SD	Mean ± SD
Giving emotional support	88.16 ± 17.5	86.11 ± 9.5
Providing safe care	93.75 ± 16.62	90.44 ± 4.26
Preventing maltreatment	78 ± 34.33	68.25 ± 2.93
Total	89.95 ± 3.54	85.47 ± 11.49

Table3 presents the items of the knowledge section in the three domains. The items with the highest scores were: “warm welcoming in entering to labor unit” (98.4%), “paying attention to safety in providing care and interventions” (97.2%) and " physical violence in the case of non-cooperation” (82.4%). The lowest knowledge score was in “freedom in choosing birthing position” (78%), “providing pain relief” (90.8%) and “attendance of unnecessary person during performing procedure” (75.2%) in the ‘giving emotional support’, ‘providing safe care’ and ‘preventing mistreatment’ domains, respectively.

Table4 shows that the items of the practice section in the three domains. The midwives reported the highest mean score practice in the items including: “I keep medical records, results of examinations and consultation confidential” (80%),“I pay attention to laboring woman’s safety in providing care and interventions” (76%) and “I do not allow the laboring woman to have companion inside the labor unit” (9.6%). The reported lowest scores in the practice section are for the items “I support laboring woman to be in desired birthing position” (42%),“I provide companions with accurate and clear information about progress of labor” (57.2%) and “I may beat the laboring woman if she does not cooperate” (38%).

### Factors related to knowledge and practice of RMC

Table5 presents overall scores of midwives’ knowledge and practice and the scores of three domains as associated with different socio-demographic characteristics.

The multivariate logistic regression model was developed to predict factors associated with RMC knowledge and practice (Table5). The odds ratios (OR) and the 95% confidence interval (95% CI) showed that among all examined socio-demographic characteristics, age had the strongest effect on midwives’ knowledge of RMC (OR = 1.116) and the work experience had the highest effect on midwives’ practice of RMC (OR = 1.118). Our findings indicate that the midwives’ knowledge and practice of RMC correspondingly increase with every additional year of their age and work experience at the birth units.

**Table 3:** Midwives’ Knowledge of Respectful Maternity Care

Domain	Item	(n = 250) n (%)
		Correct
Giving emotional support	1.Warm welcoming in entering to labor unit	246 (98.4)
	2.Showing around maternity labor unit’s environment	234 (93.6)
	3. Establishing friendly communication	242 (96.8)
	4. Encouraging and giving calming touch	217 (86.8)
	5. Calling laboring woman’s name as she desires	215 (86)
	6. Providing accurate and clear information about progress of labor, received care and interventions	238 (95.2)
	7. Providing friendly environment to ask questions	234 (93.6)
	8. Providing comfortable and calming environment	227 (90.8)
	9. Freedom in choosing birthing position	195 (78)
	10. Having companion of choice upon request	224 (89.6)
	11. Respecting laboring woman’s and her companions’ beliefs and culture	234 (93.6)
	12. Providing appropriate environment for companions	213 (85.2)
Providing safe care	13. Continuous or timely presence beside	233 (93.2)
	14. Keeping medical records and the results of tests and consultations confidential	241 (96.4)
	15. Obtaining informed consent before performing any care and interventions	228 (91.2)
	16. Providing equal care to all laboring woman regardless of their socio-economic status, ethnicity, etc.	238 (95.2)
	17. Providing evidence-based and up-to-date childbirth care	235 (94)
	18. Providing pain relief	227 (90.8)
	19. Paying attention to safety in providing care and interventions	243 (97.2)
	20. Providing accurate information about progress of labor to companions	238 (95.2)
Preventing mistreatment	21. Attendance of unnecessary person during performing procedure	62 (24.8)
	22. Physical violence in the case of non-cooperation	44 (17.6)
	23. Shouting at the laboring woman in case of non-cooperation	58 (23.2)

**Table 4:** Midwives’ Practice of Respectful Maternity Care

Domain	Item	Practice (n = 250) n (%)]				
		Always	Often	Sometimes	Rarely	Never
Giving emotional support	1. I welcome laboring woman warmly.	152 (60.8)	90 (36)	8 (3.2)	0 (0)	0 (0)
	2. I introduce myself to the laboring woman.	182 (72.8)	57 (22.8)	11 (4.4)	0 (0)	0 (0)
	3. I show the laboring woman around the labor unit.	156 (62.4)	62 (24.8)	24 (10)	5 (2)	2 (0.8)
	4. I establish friendly and appropriate relationship with the laboring woman.	151 (60.4)	83 (33.2)	12 (4.8)	4 (1.6)	0 (0)
	5. I support laboring woman by encouraging and calming touch.	125 (50)	80 (32)	37 (14.8)	7 (2.8)	1 (0.4)
	6. I use the name preferred by a laboring woman.	130 (52)	66 (26.4)	41 (16.4)	10 (4)	3 (1.2)
	7. I am continuously or timely available beside the laboring woman.	141 (56.4)	91 (36.4)	16 (6.4)	2 (0.8)	0 (0)
	8. I provide laboring woman with correct and clear information about the care, interventions and progress of labor.	158 (63.2)	68 (27.2)	20 (8)	4 (1.6)	0(0)
	9. I build friendly relationship in a way that she feels comfortable to ask her questions.	163 (65.2)	73 (29.2)	10 (4)	4 (1.6)	0 (0)
	10. I provide a comfortable environment for laboring woman.	153 (61.2)	79 (31.6)	13 (5.2)	5 (2)	0 (0)
	11. I support the laboring woman to be in her desired birthing position.	105 (42)	89 (35.6)	46 (18.4)	8 (3.2)	2 (0.8)
	12. I keep medical records and the results of examinations and consultations confidential.	200 (80)	37 (14.8)	13 (5.2)	0 (0)	0 (0)
Providing safe care	13. I cover the laboring woman’s body during examinations, using sheets.	176 (70.4)	51 (20.4)	22 (8.8)	1 (0.4)	0 (0)
	14. I perform all interventions with laboring woman’s informed consent.	177 (70.8)	59 (23.6)	10 (4)	4 (1.6)	0 (0)
	15. I provide equal care to all women, regardless of their socio-economic status, ethnicity, etc.	190 (76)	47 (18.8)	13 (5.2)	0 (0)	0(0)
	16. I support laboring woman to take care of herself and her baby.	184 (73.6)	54 (21.6)	10 (4)	2 (0.8)	0 (0)
	17. I provide evidence-based and up-to-date childbirth care.	178 (71.2)	59 (23.6)	12 (4.8)	1 (0.4)	0 (0)
	18. I pay attention to laboring woman’s safety in providing care and interventions.	190 (76)	49 (19.6)	10 (4)	1 (0.4)	0(0)
	19. I respect beliefs and culture of laboring woman and her companions.	159 (63.6)	72 (28.8)	15 (6)	4 (1.6)	0 (0)
	20. I provide companions with accurate and clear information about progress of labor.	143 (57.2)	92 (36.8)	10 (4)	5 (2)	0 (0)
Preventing mistreatment	21. I do not allow the laboring woman to have companion inside the labor unit.	24 (9.6)	23 (9.2)	46 (18.4)	116 (46.4)	41 (16.4)
	22. I may beat the laboring woman if she does not cooperate.	15 (6)	14 (5.6)	35 (14)	91 (36.4)	95 (38)
	23. I may shout at laboring if she does not cooperate.	20 (8)	16 (6.4)	28 (11.2)	96 (38.4)	90 (36)

**Table 5:** Logistic regression model of factors related to knowledge and practice of RMC among Iranian midwives

Factors	OR	95% CI	P-value
Predictive factors of knowledge Age	1.116	1.04–1.18	< 0.0001
Predictive factors of practice Work experience	1.118	1.05–1.19	0.001

## Discussion

This study demonstrated that Iranian midwives have appropriate knowledge and practice of RMC. The participating midwives had the highest score in the ‘providing safe care’ domain. This finding is not surprising because most Iranian midwives are well-trained in and have positive attitudes to physiological approaches to childbirth based on scientific evidence, and providing emotional support during labor. [25] However, the medicalized context of Iranian health system is a significant barrier to performing RMC.[21].

Furthermore, the studies on midwifery continuous models of care during labor and birth have demonstrated that midwifery-led continuity of care models result in safer care and better outcomes for mother and baby when compared to medical models of care 26, 27. Canadian midwives reported that midwives have a strong belief in vaginal birth and a desire to reduce unnecessary medical interventions, including lower Caesarean section rates. [28] In the ‘providing safe care’ domain, “paying attention to women’s safety” in knowledge section, and “I care about laboring women’s safety” in practice section had the highest scores. This indicates that Iranian midwives respect women’s right to the highest attainable level of health which is in line with the first women’s right introduced with WRA. [5].

In contrast, the items “providing pain relief” and “I provide companion with accurate information” in the “providing safe care” domain of the both knowledge and practice sections had the lowest scores respectively. While providing pain relief during labor is mentioned in Iranian guidelines for management of vaginal birth but has not been implemented in the practice yet, therefore midwives do not have enough knowledge about the pharmacological and non-pharmacological pain relief methods. Additionally, the low score of providing pain relief may be due to the inadequate resources and staffing levels. The shortage of health staff, including midwives and anesthetists may be a contributing factor to the inadequate labor pain management or providing information for a birthing companion, [29] also because it further increases workload and subsequently reduces the time that staff can allocate for pain management or providing information. [30].

In our study, participant midwives had relatively good knowledge and practice about ‘giving emotional support’ domain. In this domain, items “welcoming laboring women with respect” and “establishing friendly communication” in knowledge section had the highest scores. The importance of establishing warm and friendly relationship with childbearing women is emphasized in several studies from developed countries. [31–33] Additionally, WHO reported that laboring women who have good social support during labor and birth on average tend to have shorter labors, their pain is better controlled and there is less need for medical interventions. [32] In addition, the item “I keep medical records, results of examinations and consultation confidential” in the practice section had the highest scores. This is congruent with the finding among English and Spanish women who believed that sharing their personal information on their labor and childbirth was a sign of disrespect. [34].

In our study, in the ‘giving emotional support’ domain, midwives had the lowest scores in the item “freedom in choosing birth position” in knowledge section and item “I support laboring women to be in a desired position” in practice section. The WHO review (2018) argued that the lack of respect for women’s preferred birth position may lead to women’s undesirable experience. [6] In many parts of the world, the women are not allowed or encouraged to change their birth positions. The reasons might be due to health workers. For example, healthcare providers in Bangladesh, Cuba and Uganda lack training on birth positions and women are consequently not allowed to give birth in any position other than lying down. [35].

In contrast, Iranian midwives are trained and aware of supporting women to move and change position; however, in the medicalized context of study hospitals, midwives work under supervision of obstetricians who restrict laboring women to the bed to facilitate interventions. [36] This is in line with the findings from a systematic review published in 2012 which demonstrated the impact of workplace environment on midwifery practice, while levels of experience, knowledge and training of midwives also influence their support for maternal choice of position and mobilization during labor. [37] There is therefore a need within clinical practice to support skill development of midwives, starting during midwifery education program and providing appropriate physical structure of the labor unit.

In our study, the lowest score was obtained in the ‘preventing mistreatment’ domain. In the knowledge and practice sections, the lowest scores were obtained for the items “attendance of unnecessary person during performing procedure” and “I may beat the laboring women in the case non-cooperation” items, respectively. These items are related to women’s rights to privacy and consented care, free from harm and ill treatment [38] which should be considered during providing labor and birth care.

In this study, being older and higher work experience at the birth units resulted in higher scores in the midwives’ knowledge and practice on many of the RMC domains. After adjustment for potential confounders in the logistic regression, a clearer association emerged between midwives’ knowledge and practice, and socio-demographic factors. In the study of Dynes et al. study (2018), providers’ age was a facilitator factor for providing non-abusive behavior and being kind. Providers aged 50 years and older provided higher levels of RMC care than those in their twenties. The older providers are more experienced in providing care during labor and birth and may be more patient and less likely to treat women with aggression. [39] These findings are confirmed in our study which also demonstrated that the professionals with more working experience are more likely to be confident and self-efficacious in providing quality care in the labor unit.

Our findings demonstrated that Iranian midwives scored lower on all domains of the practice section compared to the knowledge section of the MKP-RMC. This may be due to their working environment which is over-medicalized and midwives are marginalized in providing care during labor and childbirth. [22] Midwives today have little authority to make decisions about performance of practices during labor and all caring process is managed by obstetricians. [40].

## Strengths and limitations

This survey was conducted by administrating a standardised MKP-RMC scale which has been developed using robust methods. This study was conducted in governmental (teaching/non-teaching) and semi-governmental hospitals in all regions of Tehran that is strength of this study. However, the knowledge and practice of midwives in private hospitals and other provinces of Iran may be different.

The midwives’ practice was assessed using a self-reported questionnaire, which may be a limitation of this method. However, self-reporting is an accepted way of measuring behaviors [39] and several studies confirmed its effectiveness. [41, 42] Another limitation of the present study was to inability to verify with a women’s experience measure.

## Conclusion

Iranian midwives considered preserving mothers’ and their babies’ wellbeing more important than preventing mistreatment, resulting from the importance of care provision during labor and birth in the Iranian healthcare system.

Developing a tailored educational program is recommended to enhance midwives’ knowledge and practice on promoting respectful maternity care and preventing mistreatment. Further research is needed to confirm midwives’ knowledge and practice about RMC and its determinants. In addition, an increased understanding of the association between midwives’ demographic characteristics and MKP-RMC may improve the provision of care quality during labor and birth and should be considered in the design of maternity care programs and policies. There is a need to promote the RMC Charter among both women who seek care and healthcare providers.

## Data Availability

The datasets analyzed during the current study are available from the corresponding author upon reasonable request.

## Declarations

The Written informed consent to participate was obtained from the participated midwives.

We confirm that all methods were performed in accordance with the relevant guidelines and regulations.

## Abbreviations

RMC: Respectful Maternity Care.
MKP-RMC: Midwives’ Knowledge and Practice of Respectful Maternity Care.
WRA: White Ribbon Alliance.
WHO: World Health Organization.
